# Systemic DNA/RNA heteroduplex oligonucleotide administration for regulating the gene expression of dorsal root ganglion and sciatic nerve

**DOI:** 10.1016/j.omtn.2022.05.006

**Published:** 2022-05-06

**Authors:** Hidetoshi Kaburagi, Tetsuya Nagata, Mitsuhiro Enomoto, Takashi Hirai, Masaki Ohyagi, Kensuke Ihara, Kie Yoshida-Tanaka, Satoe Ebihara, Ken Asada, Hiroyuki Yokoyama, Atsushi Okawa, Takanori Yokota

**Affiliations:** 1 Department of Orthopedic and Spinal Surgery, Graduate School of Medical and Dental Sciences, Tokyo Medical and Dental University, Tokyo, Japan; 2 Department of Neurology and Neurological Science, Graduate School of Medical and Dental Sciences, Tokyo Medical and Dental University, Tokyo, Japan; 3 Center for Brain Integration Research, Tokyo Medical and Dental University, Tokyo, Japan

**Keywords:** MT: Oligonucleotides: Therapies and Applications, antisense oligonucleotide, DNA/RNA heteroduplex, dorsal root ganglion, neuropathic pain, sciatic nerve, peripheral nerve disease

## Abstract

Neuropathic pain, a heterogeneous condition, affects 7%–10% of the general population. To date, efficacious and safe therapeutic approaches remain limited. Antisense oligonucleotide (ASO) therapy has opened the door to treat spinal muscular atrophy, with many ongoing clinical studies determining its therapeutic utility. ASO therapy for neuropathic pain and peripheral nerve disease requires efficient gene delivery and knockdown in both the dorsal root ganglion (DRG) and sciatic nerve, key tissues for pain signaling. We previously developed a new DNA/RNA heteroduplex oligonucleotide (HDO) technology that achieves highly efficient gene knockdown in the liver. Here, we demonstrated that intravenous injection of HDO, comprising an ASO and its complementary RNA conjugated to α-tocopherol, silences endogenous gene expression more than 2-fold in the DRG, and sciatic nerve with higher potency, efficacy, and broader distribution than ASO alone. Of note, we observed drastic target suppression in all sizes of neuronal DRG populations by *in situ* hybridization. Our findings establish HDO delivery as an investigative and potentially therapeutic platform for neuropathic pain and peripheral nerve disease.

## Graphical abstract

**Figure:**
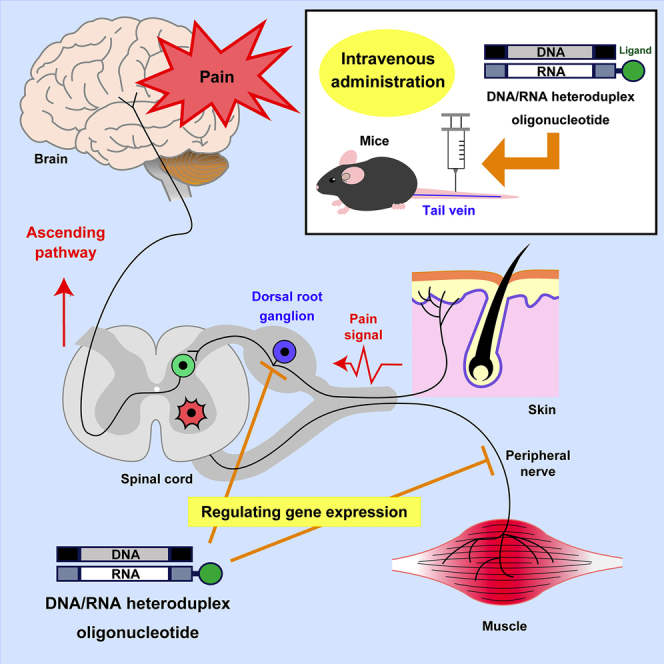

## Teaser

Kaburagi and colleagues demonstrated that a DNA/RNA heteroduplex oligonucleotide (HDO) distributed more efficiently to dorsal root ganglia and peripheral nerve with significant gene knockdown effect than the parent antisense oligonucleotides by systemic injection. This HDO technology is expected to lead to the development of gene-based therapies for neuropathic pain and peripheral nerve diseases.

## Introduction

**Figure 1:**
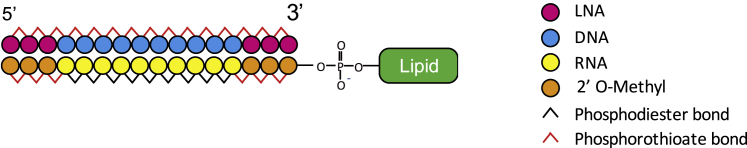
Design of ASO and Toc-HDO Schematic illustrations of ASO and Toc-HDO structure. HDO is composed of a gapmer ASO, duplexed with complementary RNA (cRNA) conjugated to delivery ligands. Gapmer ASOs have a central gap region of phosphorothioate (PS)-modified DNA flanked by nucleic acid analogs such as MOE, constrained Ethyl (cEt), or LNA, enhancing cRNA affinity.

Antisense oligonucleotides (ASOs) bind target RNAs via the Watson-Crick base pairing to regulate RNA expression. ASOs are synthetic single-stranded nucleic acids of 13–25 nt, complementary to the variable target RNA sequence. By tailoring the design, chemistry, and target RNA binding site, ASOs function through a variety of mechanisms, such as gene knockdown, splicing modulation, or miRNA inhibition. A common mechanism of selective suppression of target RNA by ASO is described below. Following the RNA/DNA heteroduplex formation, due to ASO binding to the target RNA, endogenous RNase H is recruited and then selectively degrades the target RNA. Successes in the development of ASO therapeutics for spinal muscular atrophy,^1^ Duchenne muscular dystrophy,^2^, ^3^, ^4^ or familial amyloid polyneuropathy^5^ predict a promising future for patients. Multiple clinical trials using ASO therapies for amyotrophic lateral sclerosis,^6^ Alzheimer’s disease,^7^ Parkinson’s disease,^8^ and others are ongoing. As ASO therapies selectively regulate target RNA, they can be applied to other previously untreatable or refractory diseases, such as rare diseases or cancer. We recently have developed a novel DNA/RNA heteroduplex oligonucleotide (HDO) technology that achieves highly efficient gene knockdown *in vivo*.^9^, ^10^, ^11^ HDO is composed of a gapmer ASO, duplexed with complementary RNA (cRNA) conjugated to delivery ligands. Gapmer ASOs have a central gap region of phosphorothioate (PS)-modified DNA flanked by nucleotides, enhancing cRNA affinity (Figure 1A). After internalization into the cells, the cRNA of HDO is cleaved by endogenous nucleases, resulting in the release of the ASO strand. The released ASO can now hybridize with its RNA target and promote its degradation by RNase H1-mediated cleavage.^12^^,^^13^ Intravenously (i.v.) injected α-tocopherol-conjugated HDO (Toc-HDO) enhances the potency to reduce the expression of ApoB100 mRNA by 20-fold in the liver relative to the parent.^9^

Neuropathic pain,^14^ caused by a lesion or disease of the somatosensory system,^15^ is a chronic neurological disorder that affects 7%–10% of the general population.^16^ Pharmacologic agents currently available for neuropathic pain have shown limited efficacy. In addition, unpleasant dose-dependent adverse effects are often observed.^17^ Due to limited access to these agents through the blood-brain barrier (BBB), several percentages of these agents systemically administered reach the target cells in the central nervous system. These therapies also require daily administration and affect the adherence, compliance, and/or persistence of patients since neurological pain lasts for a long duration.

The dorsal root ganglion (DRG) are located in the peripheral nervous system, between the dorsal horn of the spinal cord and peripheral nerve terminals such as the sciatic nerve. Somas of sensory DRG neurons are pseudo-bipolar neurons enwrapped by satellite glial cells, with a peripheral branch that innervates the target organ and a central branch that transduces somatosensory information to the spinal cord. Each neuron exhibits different molecular characteristics in terms of size, shape, and function, and has specific peripheral and central targets.^18^^,^^19^ Although ASOs do not cross the BBB,^14^^,^^20^^,^^21^ the DRG lacks a sufficient neurovascular barrier, enabling relatively easy access to small compounds of pharmacologic agents in the interstitium surrounding the DRG neurons.^22^ It is expected to be an important target of ASO therapies. The development of peripheral pharmacological treatments aimed at DRG tissues, primary sensory neurons, and the sciatic nerve offers more efficacious and safer treatments for inflammatory, neuropathic, cancer, and other chronic pain states. Several reports have shown amelioration of various pain symptoms with ASO or short hairpin RNA (shRNA) administration in different animal models.^14^^,^^23^, ^24^, ^25^, ^26^, ^27^, ^28^, ^29^, ^30^ However, safety, efficacy, dosage, and dose regimen were not optimized in these studies, except in one paper.^14^

Here, we report a proof-of-concept study that i.v.-injected Toc-HDO can induce a highly efficient knockdown of a target neuron in the mouse dorsal root ganglion from the cervical to lumbar cord and sciatic nerve without the influence of basal pain responses compared to the parent single-stranded gapmer ASO. This study provides a new modality for the administration of a chemically synthesized oligonucleotide that can control neuropathic pain and peripheral nerve disease.

## Results

### Gene silencing efficacy of ASO or Toc-HDO in lumbar and cervical dorsal root ganglion following systemic injection

**Figure 2:**
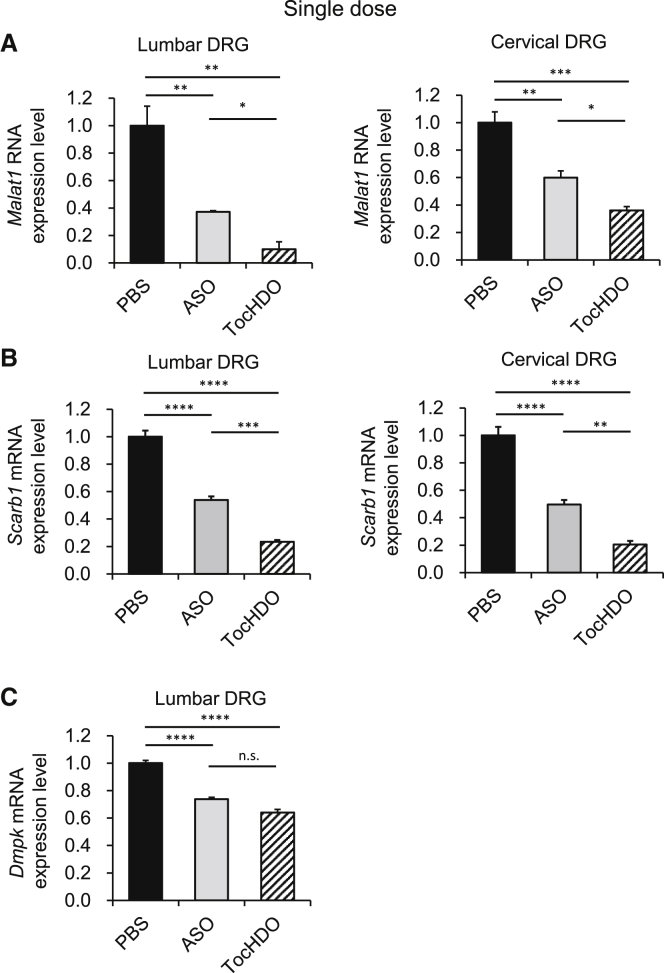
Gene silencing by single intravenous (i.v.) administration of Toc-HDO targeting endogenous genes in mouse lumbar and cervical DRG *in vivo* (A–C) Target RNA or mRNA levels measured using quantitative real-time-PCR in lumbar and cervical DRG at 72 h after i.v. injection of 50 mg/kg ASO, Toc-HDO at doses equivalent to 50 mg/kg of ASO, or PBS alone. (A) *Malat1*, (B) *Scarb1*, and (C) *Dmpk* . Data shown are relative to *Gapdh* mRNA levels and are expressed as mean values ±SEMs (n = 3–4). ∗p < 0.05, ∗∗p < 0.005, ∗∗∗p < 0.001, ∗∗∗∗p < 0.0001

**Figure 3:**
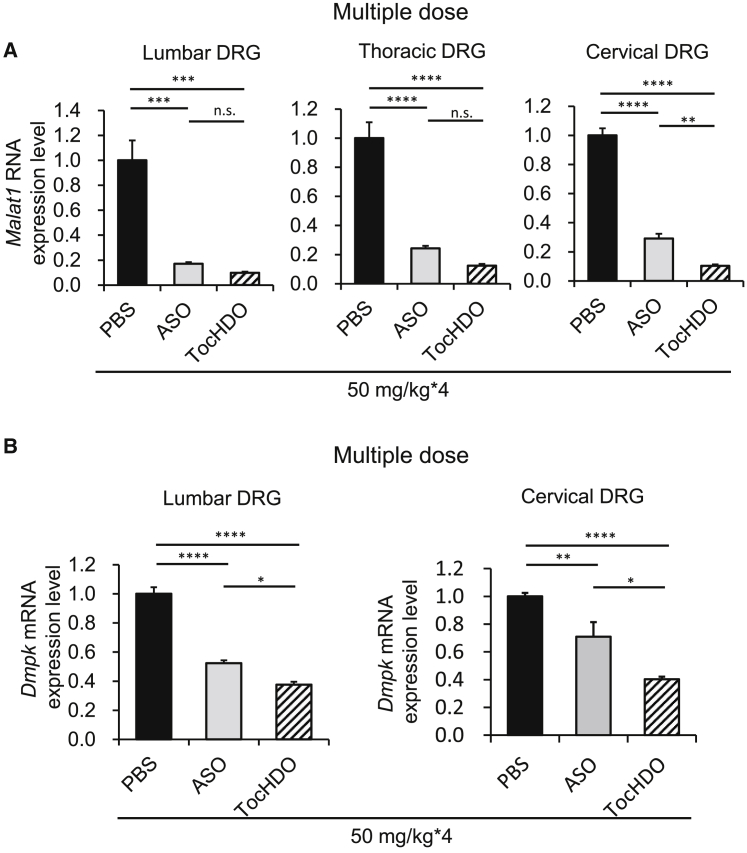
Enhanced gene-silencing effect by multiple i.v. administered Toc-HDO in mouse DRG (A) *Malat1* RNA levels measured using quantitative real-time-PCR in lumbar, thoracic, and cervical DRG at 72 h following 4 weekly i.v. injections of 50 mg/kg ASO, Toc-HDO at doses corresponding to 50 mg/kg of ASO, or PBS alone. (B) *Dmpk* mRNA levels measured using quantitative real-time-PCR in lumbar, and cervical DRG at 72 h after 4 weekly i.v. injections of 50 mg/kg ASO, Toc-HDO at doses corresponding to 50 mg/kg of ASO, or PBS alone. Data shown are relative to *Gapdh* mRNA levels and are expressed as mean values ±SEMs (n = 4/group). ∗p < 0.05, ∗∗p < 0.005, ∗∗∗p < 0.001, ∗∗∗∗p < 0.0001

We targeted a non-coding RNA gene, metastasis-associated lung adenocarcinoma transcript 1 (*Malat1*). It is also known as nuclear-enriched abundant transcript 2 (*Neat2*), originally identified in pulmonary adenocarcinoma, is among the most abundant long non-coding RNAs (lncRNAs) in the nervous system.^31^ The other two target genes were the scavenger receptor class B type 1 (*Scarb1*), a multiligand membrane receptor protein that functions as a physiologically relevant high-density lipoprotein (HDL) receptor,^32^ or dystrophia myotonica protein kinase (*Dmpk*) gene, and the expansion of the CUG repeat, which causes myotonic dystrophy type 1. This is because these sequences of ASOs demonstrate high specificity and efficacy in previous studies validated by Ionis Pharmaceuticals.^33^, ^34^, ^35^ For the HDO experiment, we designed the cRNA complementary to each ASO sequence covalently conjugated to α-tocopherol at the 5′-end (Figure 1A; Table S1). To examine the *in vivo* efficacy of Toc-HDO, we i.v. injected Toc-HDO or parent ASO targeting mouse *Malat1* at doses corresponding to 50 mg/kg of ASO. After 72 h, Toc-HDO showed a significant reduction in *Malat1* RNA expression compared with an equivalent molar of the parent ASO in the lumbar and cervical DRG (Figure 2A). A similar finding was observed using the Toc-HDO targeting mouse *Scarb1* at equimolar doses to the parent ASO (Figure 2B). A similar trend was also observed with Toc-HDO targeting mouse *Dmpk*, although the difference was not significant between ASO and Toc-HDO (Figure 2C). These results indicated that systemic administration of ASO alone had a sufficient effect on the lumbar DRG, as previously reported,^14^ and on cervical DRG. Interestingly, Toc-HDO reduced the expression of these target genes more efficiently than ASO in both the lumbar and cervical DRG. To verify these results as reliable, we i.v. multiple administered (four times weekly) with Toc-HDO or parent ASO targeted for *Malat1* or *Dmpk* at doses corresponding to 50 mg/kg of ASO. As for *Malat1* gene knockdown, we detected the enhancement of the gene knockdown effect by multiple injections of Toc-HDO or ASO compared with that seen following a single injection. More than 90% gene knockdown by Toc-HDO was observed throughout the spinal cord, which is greater than that by ASO (induced by 75% knockdown; Figure 3A). Although the knockdown effect of ASO diminished from the lumbar DRG to the cervical DRG, Toc-HDO was not attenuated. A similar result was observed by Toc-HDO targeting *Dmpk* in the lumbar and cervical DRG and no attenuation of knockdown in cervical DRG (Figure 3B). As shown in Figure S1, Toc-HDO (20-mer) using MOE/DNA gapmer targeting mouse *Malat1* also showed superior gene suppression compared to ASO at a dose of 50 mg/kg in lumbar DRG. These results suggest that systemic administration of Toc-HDO downregulated the target gene of interest predominantly in the DRG *in vivo* compared to ASO.

Similar data were obtained from DRG of mice that were i.v. administered (12.5 mg/kg; 4 times weekly) with parent ASO or Toc-HDO at the same molar dose to ASO targeted for *Malat1* (Figure S2). Four repeated doses of Toc-HDO and ASO showed RNA reduction in the cervical DRG. The efficacy was almost equivalent to that of a single administration of 50 mg/kg Toc-HDO and ASO, but not lumbar DRG. RNA reduction by a single dose of 50 mg/kg Toc-HDO was obviously better than that by 4 repeated doses of 12.5 mg/kg Toc-HDO in the lumbar DRG.

### Toc-HDO or ASO suppresses target RNA in DRG in a dose-dependent manner

**Figure 4:**
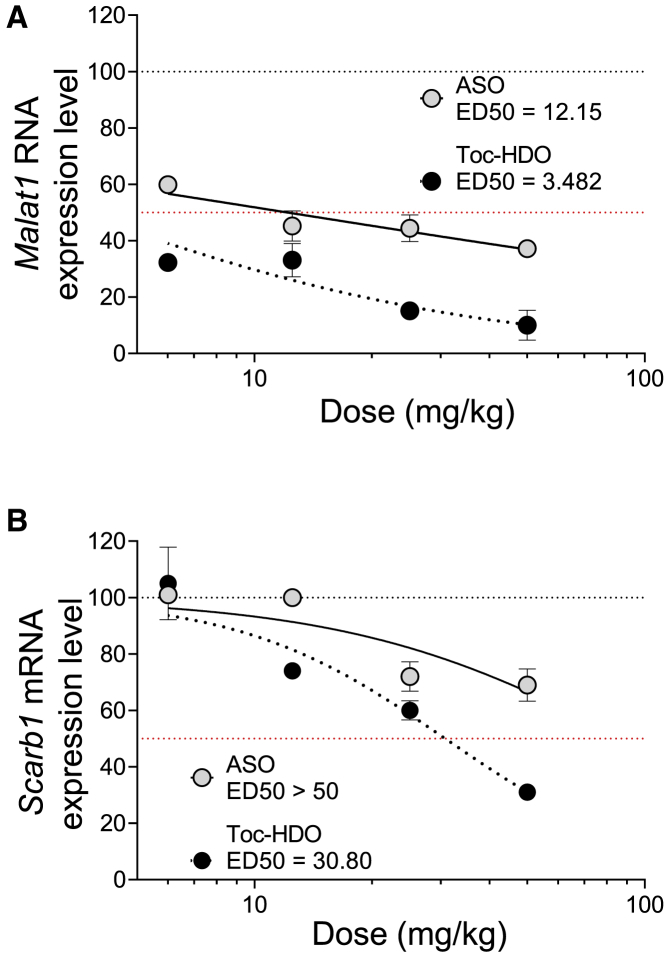
Dose-dependent reduction of *Malat1* RNA and *Scarb1* mRNA levels in lumbar and cervical DRG following i.v. injection of PBS, ASO, or Toc-HDO (A) *Malat1* RNA levels were measured using quantitative real-time-PCR in lumbar DRG at 72 h following i.v. injection of ASO, Toc-HDO, or PBS alone at different doses (6.25, 12.5, 25, or 50 mg/kg) (n = 3). (B) *Scarb1* mRNA levels were measured using quantitative real-time-PCR in lumbar tissues at 72 h after i.v. injection of ASO, Toc-HDO, or PBS alone at different doses (6.25, 12.5, 25, or 50 mg/kg) (n = 3).

To examine the pharmacodynamic activity of Toc-HDO in DRG, ascending doses of Toc-HDO or ASO targeting Malat1 RNA or *Scarb1* mRNA were injected i.v. into mice. In DRG, dose-dependent target suppression of ASO or Toc HDO was attained with an R^2^ value > 0.50, as shown in Table S2. Toc-HDO targeting *Malat1* RNA in the lumbar DRG was more efficient than the parent ASO. The 50% effective dose (ED_50_) in the lumbar DRG was 3.48 mg/kg, 3.5-fold more potent than that of the ASO, the ED_50_ of which was 12.15 mg/kg (Figure 4A). The ED_50_ of Toc-HDO targeting *Scarb1* in lumbar DRG was 30.80 mg/kg (Figure 4B). However, the ED_50_ of ASO targeting *Scarb1* in the lumbar or cervical DRG was >50 mg/kg (Figure 4B).

### Toc-HDO and ASO exhibit long-standing activity

**Figure 5:**
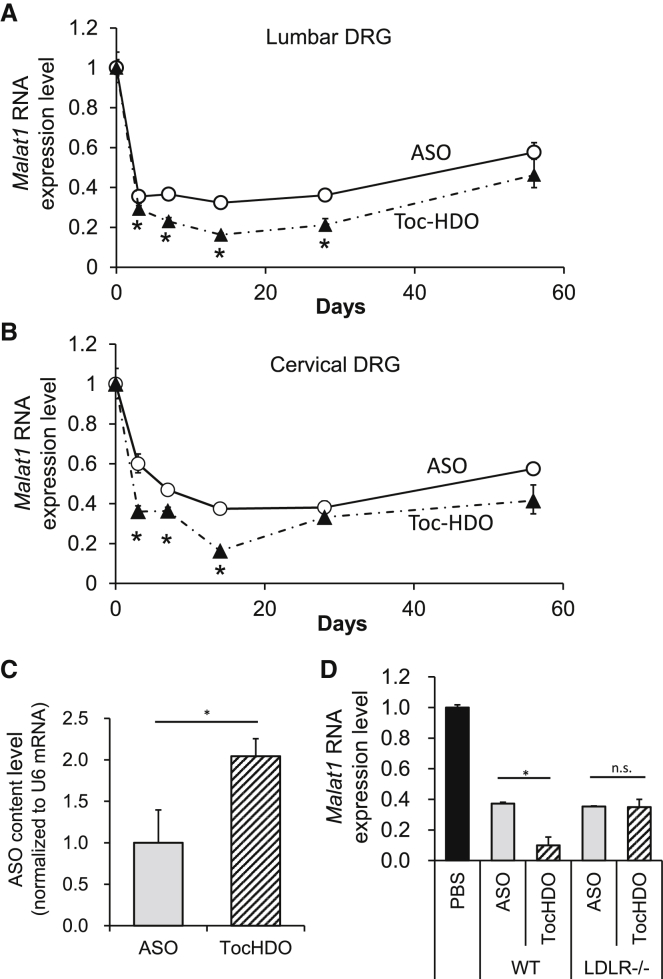
Gene-silencing duration following Toc-HDO administration in lumbar DRG (A) Quantitative real-time-PCR analyses of *Malat1* RNA levels in the lumbar DRG at the indicated time points after injection of 50 mg/kg ASO, Toc-HDO at doses corresponding to 50 mg/kg of ASO, or PBS alone. (B) Quantitative real-time-PCR analyses of *Malat1* RNA levels in cervical DRG at the indicated time points after injection of ASO, Toc-HDO at doses corresponding to 50 mg/kg of ASO, or PBS alone. (C) Quantification of the parent ASO strand after 4 weekly administrations of ASO or Toc-HDO. The amount of the parent ASO strand in the DRG was measured using quantitative real-time-PCR. (D) Quantitative real-time-PCR analyses of *Malat1* RNA levels in lumbar DRG after injection of 50 mg/kg ASO, Toc-HDO at doses corresponding to 50 mg/kg of ASO, or PBS alone into WT or LDLR knockout mice (n = 4/group). ∗p < 0.05, ∗∗p < 0.005, ∗∗∗p < 0.001, ∗∗∗∗p < 0.0001

We then tested the duration of gene-silencing efficacy in the lumbar and cervical DRG following a single administration of Toc-HDO or ASO targeting *Malat1*. The reduction in *Malat1* RNA in both DRG by Toc-HDO and ASO was maximal on day 14 (T_max_) and lasted for at least more than 2 months (Figures 5A and 5B). Surprisingly, the superiority of Toc-HDO compared with ASO also lasted for more than 1 month in the lumbar DRG or for 2 weeks in the cervical DRG (Figures 5A and 5B). Next, we analyzed the amount of i.v. administered Toc-HDO or ASO that was distributed to the DRG after four repeated i.v. injections. Quantitative real-time-PCR was used to quantify the ASO strands in the RNA samples extracted from the DRG. Accumulation of ASO in the DRG was twice as high in mice injected with Toc-HDO than in mice injected with ASO (Figure 5C). To examine the uptake mechanism by DRG, we injected Toc-HDO into Ldlr^−/−^ mice, as previously reported.^9^ The suppression of target Malat 1 mRNA was decreased in Ldlr^−/−^ mice to the same level as that of the parent ASO (Figure 5D). This indicated that uptake of Toc-HDO by DRG was mediated, at least in part, through the low-density lipoprotein (LDL) receptor, similar to the Toc-HDO uptake by the liver.^9^

### Gene-silencing efficacy of ASO or Toc-HDO in the sciatic nerve after systemic injection

**Figure 6:**
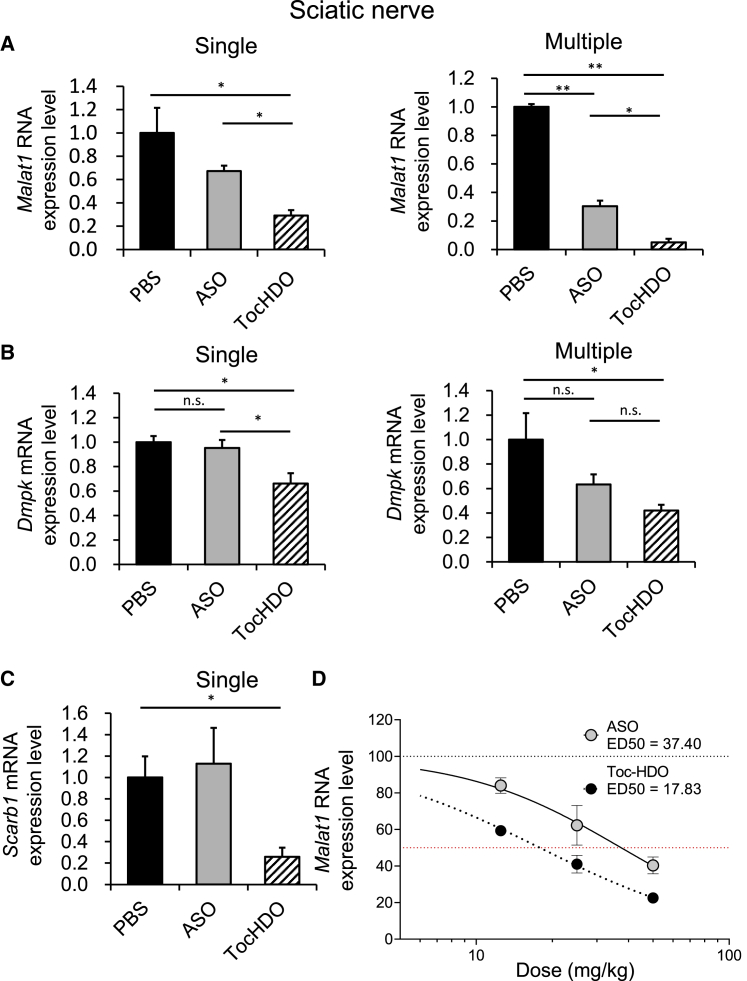
Gene-silencing effect by single or multiple i.v. administered Toc-HDO in mouse sciatic nerve (A) *Malat1* RNA levels measured using quantitative real-time-PCR in the sciatic nerve of mice after single or 4 weekly i.v. injections of 50 mg/kg ASO, Toc-HDO at doses corresponding to 50 mg/kg of ASO, or PBS alone. (B) *Dmpk* mRNA levels in the sciatic nerve after single or 4 weekly i.v. injections of 50 mg/kg ASO, Toc-HDO at doses corresponding to 50 mg/kg of ASO, or PBS alone. (C) *Scarb1 m* RNA levels in the sciatic nerve after single injections of 50 mg/kg ASO, Toc-HDO at doses corresponding to 50 mg/kg of ASO, or PBS alone. Data shown are relative to *Gapdh* mRNA levels and are expressed as mean values ±SEMs (n = 4). (D) Dose-response curve showing *Malat1* RNA knockdown in the sciatic nerve after a single i.v. injection of PBS, ASO, or Toc-HDO at different doses (12.5, 25, or 50 mg/kg, 4/group). The sciatic nerves were harvested 3 days after dosing. ∗p < 0.05, ∗∗p < 0.005, ∗∗∗p < 0.001, ∗∗∗∗p < 0.0001

We also evaluated the gene knockdown in the sciatic nerve, which is composed of axons projecting from sensory neurons residing in lumbar DRG, after single or multiple systemic injections. After 72 h, both Toc-HDOs with locked nucleic acid (LNA) wing or 2′-*O*-methoxy-ethyl (MOE) wing showed a significant reduction in *Malat1* RNA expression compared with that seen in an equivalent molar of the parent ASO in the sciatic nerve (Figures 6A and S1). Similar findings were observed using Toc-HDO targeting mouse *Scarb1* and *Dmpk* mRNA (Figures 6B and 6C). In the sciatic nerve, dose-dependent target suppression of ASO or Toc HDO was also achieved. Toc-HDO targeting Malat1 RNA in the sciatic nerve was more efficient than the parent ASO. The ED_50_ was 17.83 mg/kg, 2.1-fold more potent than that of the ASO (Figure 6D). These results indicated that Toc-HDO also reduced the expression of these target genes more efficiently than did ASO in the sciatic nerve.

### Distribution of the target RNA reduction by *in situ* hybridization and oligonucleotides using anti-PS antibody staining

**Figure 7:**
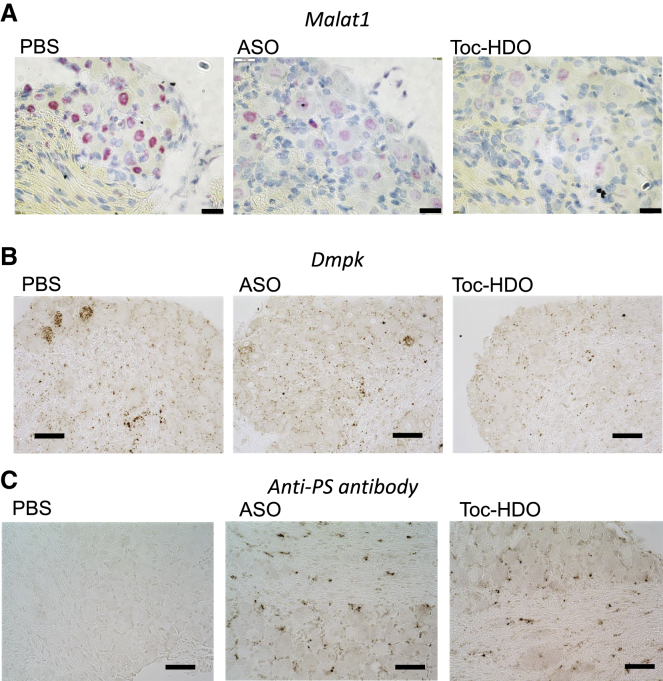
Distribution of target RNA reduction by *in situ* hybridization and oligonucleotides using anti-PS antibody staining (A) *In situ* hybridization using the *Malat1* probe in the lumbar DRG after single injections of PBS, 50 mg/kg of ASO, or Toc-HDO at doses corresponding to 50 mg/kg ASO. Scale bar, 200 μm. (B) *In situ* hybridization using *Dmpk* probe in the lumbar DRG from mice 3 days following a single injection of PBS, 50 mg/kg of ASO, or Toc-HDO at doses equivalent to 50 mg/kg of ASO. Scale bar, 50 μm. (C) Immunohistochemistry staining by anti-PS antibody in lumbar DRG from mice 3 days after the injection of 50 mg/kg of ASO, Toc-HDO at doses equivalent to 50 mg/kg of ASO against *Dmpk* , or PBS alone. Scale bar, 50 μm.

To evaluate the distribution of the target RNA reduction, we performed an *in situ* RNA hybridization analysis of *Malat1* in the lumbar DRG tissues from mice with single injections of 50 mg/kg parent ASO, Toc-HDO at doses corresponding to 50 mg/kg of ASO, or phosphate-buffered saline (PBS) alone. *Malat1* signal intensity obtained using ViewRNA was observed in the nuclei of small, medium, and large DRG neurons, but not in the DRG treated with PBS. Dramatic *Malat1* reduction was observed in all three sizes of neurons in the lumbar DRG from mice treated with Toc-HDO, which is consistent with the quantitative real-time-PCR results. Alternatively, the decreased reduction was consistently observed in mice treated with ASO (Figures 6A and 7A). In axon bundles, a few *Malat1* signal intensities were observed in PBS-treated lumbar DRG and completely disappeared after Toc-HDO treatment. To detect the target mRNA expression more sensitively, we evaluated the *in situ* RNA hybridization analysis of *Dmpk* by RNAscope in the lumbar DRG tissues from mice after single injections (Figure 7B). In contrast to the distribution of *Malat1* signal distribution, Dmpk signal intensity was observed in several nuclei of DRG neurons with a diameter of approximately 30 μM and the interstitium surrounding the DRG neurons. After Toc-HDO injection, *Dmpk* reduction was observed in both DRG neurons with a diameter of approximately 30 μM and the interstitium surrounding the DRG neurons. To investigate whether relative potency correlated with ASO distribution (the kinetic distribution), the DRG in mice treated with a single administration of Toc-HDO or ASO targeting *Dmpk* were also sectioned and stained with a pan anti- PS polyclonal antibody.^36^ Anti-PS signal intensity was observed diffusely in the cytoplasm and surrounding interstitium of DRG neurons treated with Toc-HDO (Figure 7C). Alternatively, fewer signals were observed in the DRG of mice treated with ASO (Figure 7C).

### ASO or Toc-HDO do not affect pain behavior

To investigate whether Toc-HDO or ASO does not influence basal pain responses due to nonspecific effects,^14^ mice were injected i.v. 4 times weekly with 50 mg/kg ASO, Toc-HDO at doses corresponding to 50 mg/kg ASO, or PBS alone, and responses to noxious and non-noxious mechanical forces applied to the hind feet were monitored for 4 weeks after the last injection. Measurement of tactile allodynia by von Frey testing revealed that Toc-HDO or ASOs did not alter paw withdrawal thresholds compared with that of PBS-treated mice (Figure S3B). We next assessed the effects of ASOs on acetone-evoked pain behaviors to determine whether cold allodynia was observed in the presence of Toc or ASO (Figure S3C). Finally, to measure differences in thermal thresholds, we performed a hot plate test and observed no significant difference in the latency for licking the hind paw (Figure S3D). Collectively, these data suggested that Toc or ASOs did not affect basal pain responses and were not activated. We then evaluated the serum chemistry of Toc-HDO and ASO. Serum chemistry analyses and liver and kidney histopathology did not show any abnormalities following single or multiple administrations of Toc-HDO or ASO targeting *Malat1* (Figure S4; Table S3).

## Discussion

We demonstrated that Toc-HDO targeting endogenous genes was efficiently delivered into the mouse DRG and sciatic nerve with i.v. administration and induced a significant reduction of target gene expression compared to the parent ASO. Toc-HDO also showed target gene knockdown in sensory neurons in the DRG by *in situ* hybridization. Its suppressive effect showed whole cervical to lumbar DRG, cumulative and long-lasting, suggesting that further optimization of dosing regimen delivers the full potential of HDO technology. Given that this is a proof-of-concept study, we consider Toc-HDO a reasonable platform technology for modulating the *in vivo* DRG function and sciatic nerve.

Technologies regulating the *in vivo* DRG function and sciatic nerve would be beneficial for a variety of basic and clinical studies. In particular, they can advance our understanding of the biology and pathophysiology of the DRG. Associated blocking antibodies delivered systemically can then be used to inhibit some specific proteins expressed, but they only recognize the external portion of proteins on the surface of cell membranes, such as receptors, transporters, and ion channels. In contrast, ASO can also modulate the expression of proteins on the cell surface and intracellular proteins or RNA associated with cell signaling of biological and pathological processes that cause pain. Therefore, Toc-HDO can be an invaluable technology for cultivating basic studies relevant to the DRG.

As a result of examining the gene knockdown effect with three target genes, the suppression effect of Toc-HDO was significantly higher than that of parent-ASO in DRG neurons. The ED_50_ of Toc-HDOs were also significantly lower than that of the parent ASO. Thus, we observed improvements in efficacy and potency (ED_50_) in DRG at a dose of 50 mg/kg following Toc-HDO injection. Here, we also showed that Toc-HDO and ASO have a gene knockdown effect regardless of the size of these DRG neurons by *in situ* hybridization. However, it should be noted that this *Malat1* sequence represses genes very efficiently and safely compared to other target gene sequences.

DRG is exceptional in that it is highly permeable to allow easy access to a variety of low- and high-molecular-weight compounds compared with other nerve tissues. This can be explained by the DRG possessing microvessels with fenestrated endothelia and a permeable connective tissue capsule.^37^^,^^38^ However, there is a diffusion barrier within the multilayered perineurium formed by tight junctions between the neighboring perineurial cells such as glia and basement membranes, with the innermost layers preventing trans-perineurial movement of larger molecular weight tracers.^39^ The vascular permeability of the DRG has been studied using various animals and tracers in the intact and injured DRG. Despite mixed opinions on the size of molecules that can penetrate the DRG,^37^, ^38^, ^39^, ^40^, ^41^, ^42^ fluorescein isothiocyanate (FITC)-labeled dextran (70 kDa), FITC-labeled albumin (66 kDa), and horseradish peroxidase (44 kDa), which do not cross the healthy BBB or blood-nerve barrier (BNB), can gain access to the DRG. Here, we provide evidence for the access of Toc-HDO or ASO activity to sensory neurons in the DRG using *in situ* hybridization and anti-PS antibody staining.

Tissue concentrations of parent ASO and knockdown effect from DRG in normal mice injected with Toc-HDO were more than twice that of those injected with ASO. However, the gene knockdown effect of *Malat1* RNA by Toc-HDO in DRG was decreased in Ldlr^−/−^ mice to the same level as that of ASO. The LDL receptor was localized on both primary cultured neurons and the other cells dissociated from the DRG in rabbits and rats.^43^ DRG neurons expressed LDL receptor (LDLR) on both cell bodies and neurites and internalized rhodamine-labeled lipoproteins isolated from rats as well as LDL from human serum.^44^ Collectively, conjugation with α-tocopherol results in the binding of Toc-HDO to serum lipoproteins such as LDL or HDL and efficient delivery to the DRG via the LDLR.^45^ In addition, the uptake of lipoprotein via the LDLR into sensory neurons in the DRG is stimulated following axonal injury.^44^ This suggests that Toc-HDO uptake may be enhanced in the presence of nerve damage.

We often encountered patients suffering from neuropathic pain of the neck or upper limb as well as lumbar or lower limb in clinical practice. The effect of ASO on cervical DRG has not been studied.^14^ Here, we showed that ASO and Toc-HDO had gene knockdown effects in the cervical DRG; however, these were less effective than those in the lumbar DRG following a single injection. Interestingly, multiple injections of Toc-HDO showed no difference in gene knockdown effects between the lumbar and cervical DRG. Contrastingly, multiple injections of ASO remained less effective in the cervical DRG than in the lumbar DRG.

In summary, we efficiently silenced a target molecule expressed in the DRG and sciatic nerve by i.v. administration of Toc-HDO without any behavioral abnormalities. Knockdown of the target was verified at the mRNA level, and *in situ* hybridization and distribution of ASO localization were demonstrated. We believe that our platform technology based on HDO will advance a variety of basic and clinical studies on the biology and pathophysiology of the DRG and peripheral nerve. Our findings can also provide valuable therapeutic options for patients suffering from neuropathic pain and peripheral nerve diseases.

## Materials and methods

### Oligonucleotide synthesis

All chemically modified oligonucleotides for the experiment were purchased from GeneDesign (Osaka, Japan).

### Animals

All of the animal studies were approved by the Institutional Animal Care and Use Committee of the Tokyo Medical and Dental University (Tokyo, Japan). C57BL/6 mice aged 6–7 weeks (Oriental Yeast, Tokyo, Japan) or B6.129S7-Ldlr (tm1Her)/J (Ldlr^−/−^) mice (The Jackson Laboratory, Bar Harbor, ME, USA) aged 6 weeks were maintained on a 12-h light/dark cycle in a pathogen-free animal facility with free access to food and water. All of the mice were sacrificed under isoflurane anesthesia (Wako, Tokyo, Japan). After perfusion with PBS, lumbar DRG (L3/4/5), thoracic DRG (Th10/11/12), cervical DRG (C3/4/5), the liver, kidney, and sciatic nerve were removed and fixed in 10% neutral-buffered formalin solution (Wako, Tokyo, Japan) for histological analyses (RNAview, RNAscope, or anti-PS antibody staining). Serum samples were also collected.

### ASO and Toc-HDO administration

PBS or nucleic acid was i.v. injected from the tail vein, based on body weight (e.g., 50 mg/kg of body weight). Briefly, ASO or Toc-HDO was dissolved in PBS and prepared, and animals were dosed as previously described.^46^^,^^47^

### Blood chemistry and complete blood count analysis

Blood chemistry was assessed in the SRL Laboratory (Tokyo, Japan), and the blood cell count was measured using LSI Medicine (Tokyo, Japan).

### RNA isolation and quantitative real-time PCR

Total RNA was extracted from mouse tissues using the MagNA Pure 96 system (Roche Diagnostics, Mannheim, Germany). To detect mRNA, DNase-treated RNA (2 μg) was reverse transcribed using the Transcriptor Universal cDNA Master (Roche Diagnostics) according to the manufacturer’s instructions. To estimate mRNA expression and detect short oligonucleotides, including the parent ASO, quantitative real-time-PCR analysis was conducted using the Light Cycler 480 Real-Time PCR Instrument (Roche Diagnostics). The primers and probes for mouse Malat1 RNA (NR_002847.3), Dmpk mRNA (NM_016741.2), Scarb1 mRNA (NM_016741), and Gapdh mRNA (4352932E) for real-time normalization were designed by Applied Biosystems (Sciex, Framingham, MA, USA). All qPCR studies were conducted in accordance with the minimum information for publication of quantitative real-time PCR experiment guidelines.^48^ Relative ASO amounts were calculated in comparison with U6 RNA levels, which were used as internal controls, using the same method as previously published.^9^^,^^10^^,^^49^

### Immunohistochemistry of anti-PS antibody

Slides were deparaffinized in xylene, pretreated for antigen retrieval using proteinase K (Dako, Santa Clara, CA, USA), and incubated at room temperature (25°C) for 5 min. The samples were incubated in BLOX ALL (Vector Laboratories, Burlingame, CA, USA) for 10 min, to block endogenous peroxidase activity. Slides were blocked using Background Buster (Innovex Biosciences, Richmond, CA, USA) for 30 min. A polyclonal rabbit anti-ASO antibody,^36^ supplied by Ionis Pharmaceuticals, was then applied to slides at a dilution of 1:10,000 (diluted in 10% normal goat serum), and the slides were incubated at room temperature (25°C) for 1 h. After three washes in PBS, the slides were incubated with goat anti-rabbit horseradish peroxidase-conjugated secondary antibody (Jackson ImmunoResearch Laboratories, West Grove, PA, USA) at a 1:200 dilution for 30 min and then developed with 3,3′-diaminobenzidine (DAB) (Thermo Fisher, Waltham, MA, USA) and counterstained with hematoxylin.

### *In situ* hybridization using ViewRNA assay or RNAscope

Malat1 expression was detected using the QuantiGene View RNA Tissue Assay (Affymetrix, cat. no. QVT0011) according to the manufacturer’s instructions, with optimal conditions of boiling in Affymetrix pretreatment solution and protease digestion. Species-specific MALAT1 probes were purchased from Affymetrix (cat. no. VB-11110-01). Briefly, mouse tissues were fixed in 10% neutral-buffered formalin, embedded in paraffin, and sectioned into 5-μm sections. After deparaffinization, the tissue slides were boiled in Affymetrix pretreatment solution for 30 min, followed by treatment with protease at 40°C for 20 min, depending on the tissue. The Malat1 RNA probe was used at a 1:40 dilution and incubated with the sample at 40°C for 120 min. After washing, the Malat1 RNA/probe complex was hybridized with a preamplifier, amplifier, and alkaline phosphatase (AP)-oligonucleotides at 40°C. The dilution of the preamplifier and amplifier was as recommended by Affymetrix,^33^ followed by counterstaining with hematoxylin.

Dmpk expression was detected using the RNAscope 2.5 HD Brown Chromogenic Reagent Kit according to the manufacturer’s instructions (Advanced Cell Diagnostics [ACD], Westminster, CO, USA). Formalin-fixed paraffin embedded brains were sectioned (5 μm) and placed on SuperFrost Plus slides (Thermo Fisher Scientific, Waltham, MA, USA). Slides were baked in oven for 1 h at 60°C, incubated twice in xylene for 5 min, and washed in 100% ethanol twice for 1 min. Sections were incubated in hydrogen peroxide for 10 min at room temperature (25°C), washed with distilled water, and boiled at 1X RNAscope target retrieval buffer for 15 min. Slides were then washed in distilled water and transferred to 100% ethanol for 3 min and allowed to dry. Protease Plus treatment was applied to the tissues and incubated in an oven at 40°C for 30 min and washed in distilled water twice for 2 min. Slides were incubated with Dmpk probes (ACD, cat. no. 530741) for 2 h at 40°C. Further amplification of the target probe signal was performed the according to the manufacturer’s instructions (RNAscope 2.5 HD detection protocol Amp 1-6) (cat. no. 322310). The hybridization signals were detected with DAB staining.

### von Frey testing

Mechanical sensitivity was measured by applying a series of calibrated von Frey filaments (0.02–4 g) to the plantar aspect of the hind paw. Each filament was applied once to each mouse. Beginning with the 1-g filament, each filament was applied perpendicular to the hind paw for 4–6 s. A brisk withdrawal of the hind paw indicated a positive response, and a lack of withdrawal indicated a negative response. The filament testing was repeated two times, and at least two responses to the filament out of the three trials indicated an overall positive response. If the mouse demonstrated an overall positive response, then the filament with the next-lower force was applied as described above. If no overall positive response was observed (0/3 or 1/3 responses), then the filament with the next-higher force was applied as described above. Once the threshold was determined (i.e., from response to no response, or vice versa) the responses to the next five filaments were recorded to determine the median withdrawal threshold.

### Acetone response

To assess the sensitivity to cool temperatures, 100 μL acetone was applied to the plantar surface of the hind paw. As acetone evaporates, it produces a cooling sensation.^50^ Acetone was applied 5 times to each paw at an interval of at least 30 s, and the number of brisk foot withdrawals in response to the acetone application was recorded.

### Thermal response latency

The responses to noxious heat were estimated using a hot plate (NISSIN, Saitama, Japan). To measure the latency of paw flinching, licking, or withdrawal, the mice were placed in a transparent plastic chamber on a 50°C metal hot plate. To prevent tissue damage, a maximum cutoff of 30 s was used. A 5-min interval between consecutive stimulations of the same hind paw was used. The evaluation was performed three times on the left lateral plantar hind paw, and withdrawal latencies were calculated.

### Stride of hindlimb

The animals were placed in a circular pen (diameter of 1.5 m), and the left hindlimb was assessed over 5 min. The average score for the left hindlimb was determined for each animal.

### Motor function video analysis

We recorded the video of the hind paw through plastic cases as motor evaluation and measured toe spread (TS). TS is the distance from the first to fifth toe.

### Statistical analysis

GraphPad Prism 8 software and Microsoft Excel (Microsoft, Redmond, WA, USA) were used to analyze the data. All of the numerical values are presented as means ± SEMs. Differences among more than three groups were analyzed by one-way analysis of variance. Statistical differences between two groups were analyzed by Student’s one-tailed t test. (∗p < 0.05, ∗∗p < 0.005, ∗∗∗p < 0.001, ∗∗∗∗p < 0.0001).

## Supplemental information

Document S1. Figures S1–S4 and Tables S1–S3

Document S2. Article plus supplemental information
