# Supplementary material for: Systemic DNA/RNA heteroduplex oligonucleotide administration for regulating the gene expression of dorsal root ganglion and sciatic nerve
Source: Mol Ther Nucleic Acids. 2022 May 6;28:910–9. doi: 10.1016/j.omtn.2022.05.006 (PMC9167871; doi:10.1016/j.omtn.2022.05.006)
Supplement: Document S1. Figures S1–S4 and Tables S1–S3 [file mmc1.pdf]

OMTN, Volume *28*

Supplemental information

Systemic DNA/RNA heteroduplex oligonucleotide administration for regulating the gene expression of dorsal root ganglion and sciatic nerve

Hidetoshi Kaburagi, Tetsuya Nagata, Mitsuhiro Enomoto, Takashi Hirai, Masaki Ohyagi, Kensuke Ihara, Kie Yoshida-Tanaka, Satoe Ebihara, Ken Asada, Hiroyuki Yokoyama, Atsushi Okawa, and Takanori Yokota

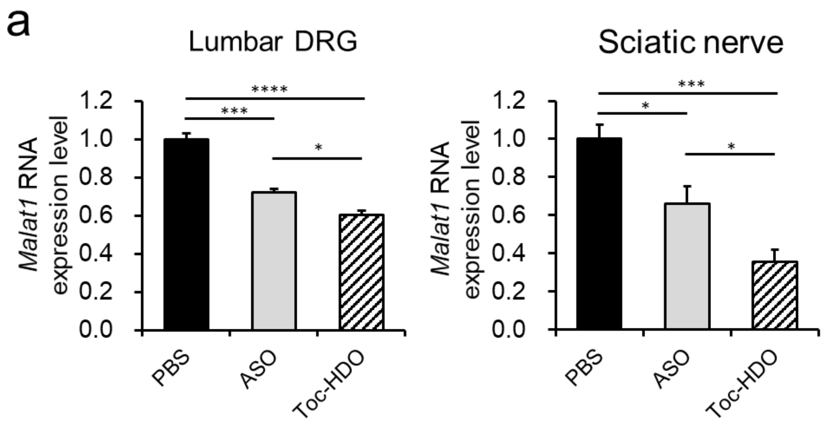

Figure S1. Gene silencing effect by intravenously administered ASO or Toc-HDO with MOE wing. (a) *Malat1* RNA levels measured using quantitative RT-PCR in lumbar DRG and sciatic nerve at 72 h after four intravenous injections of 50 mg/kg ASO, Toc-HDO with MOE wing, or PBS alone.

a

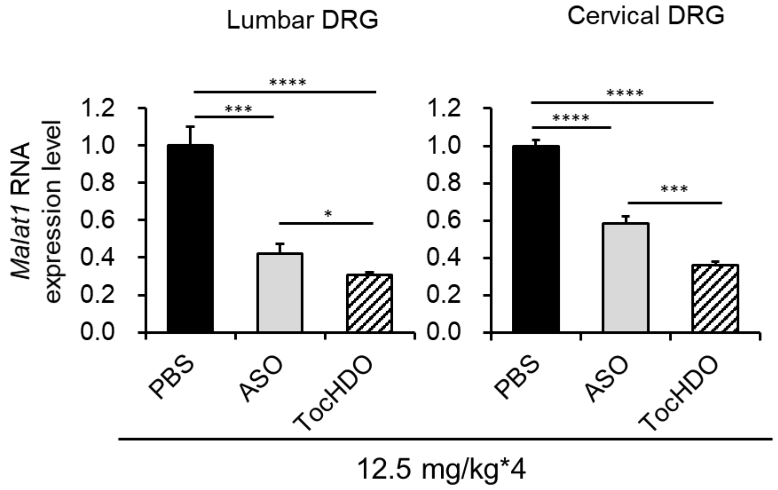

Figure S2. Enhanced gene silencing effect by multiple intravenously administered low dose of Toc-HDO in mouse DRG. (a) *Malat1* RNA levels measured using quantitative RT-PCR in lumbar and cervical DRG at 72 h after four weekly intravenous injections of 12.5 mg/kg ASO, Toc-HDO, or PBS alone.

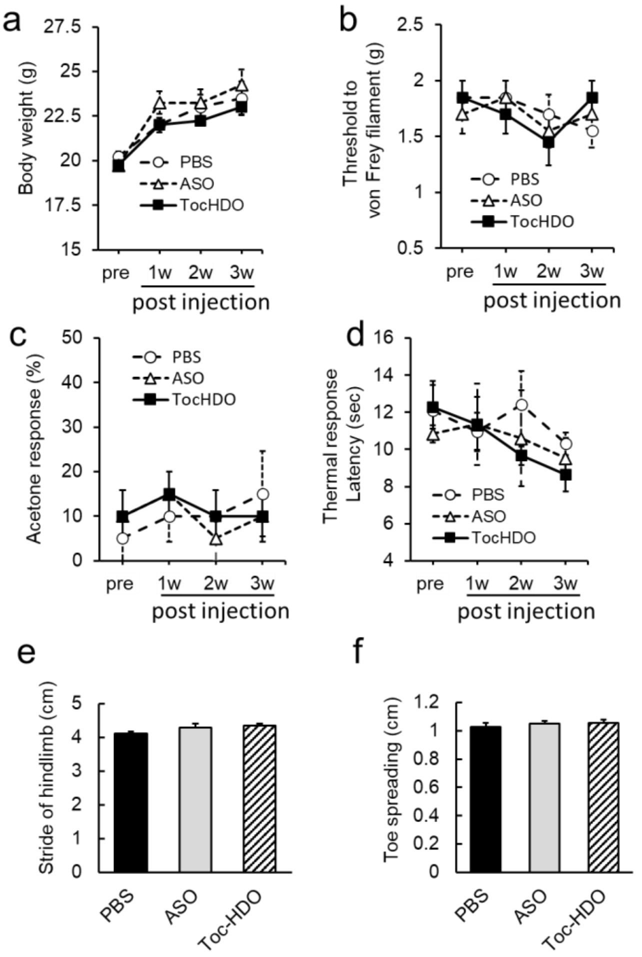

Figure S3. ***HDOs and ASOs do not modulate baseline pain behavior.***

(a) Time course of body weight following a single 50 mg/kg intravenous bolus injection of PBS, *Malat1* ASO, or *Malat1* Toc-HDO (n = 4/group). Over the 4-week experimental period, body weight remained similar in the three groups. Data are presented as the mean ± SEM **(b)** Time course of withdrawal threshold for von Frey filaments (g) following a single intravenous bolus injection of PBS, *Malat1* ASO, or *Malat1* Toc-HDO (n = 4/group). **(c)** Time course of response to acetone following a single intravenous bolus injection of PBS, *Malat1* ASO, or *Malat1* Toc-HDO (n = 4/group). **(d)** Time course of thermal response latency (s) to 50°C heat stimulation following a single intravenous bolus injection of PBS, *Malat1* ASO, or *Malat1* Toc-HDO (n = 4/group). **(e)** Distance duration dynamics of hindlimbs hours after a single intravenous bolus injection of PBS, *Malat1* ASO, or *Malat1* Toc-HDO (n = 4/group). **(f)** Measurement of toe spreading through the plastic case as motor measured toe spread after a single intravenous bolus injection of PBS, *Malat1* ASO, or *Malat1* Toc-HDO (n = 4/group).

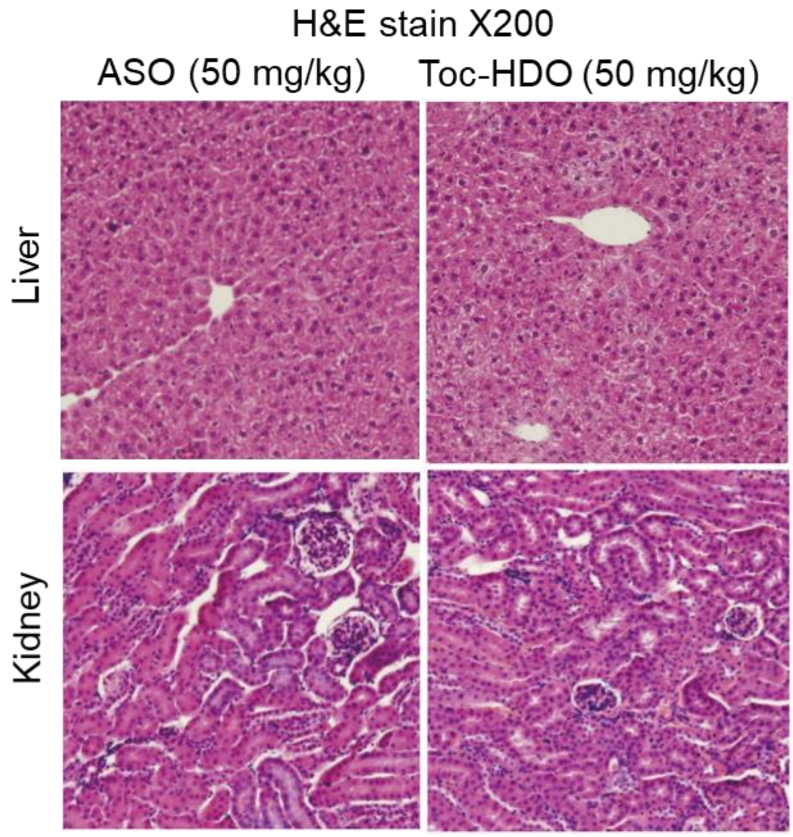

Figure S4. Liver and kidney histology. (**a**) Hematoxylin and eosin staining of the liver and kidney following four weekly 50 mg/kg injections of ASO or Toc-HDO.

Table S1. Sequences of antisense oligonucleotide and Tocopherol conjugated complementary RNA

	Target name	Sequence	Structure
ASO	*Malat1*	5′ -CTAGTTCACTGAATGC-3′	Ss
Toc-HDO	*Malat1*	5′ - CTAGTTCACTGAATGC -3<br>3′ - GAU*CAAGUGACUU*ACG-Toc-5′	Ds
ASO	*Dmpk*	5′ -ACAATAAATACCGAGG-3′	Ss
Toc-HDO	*Dmpk*	5′ -ACAATAAATACCGAGG-3′<br>3′ - TGT*UAUUUAUGGC*TCC-Toc-5′	Ds
ASO	*Scarb1*	5′ -TCAGTCATGACTTC-3′	Ss
Toc-HDO	*Scarb1*	5′ -TCAGTCATGACTTC-3′<br>3′ - AG*UCAGUACUGA*AG-Toc-5′	Ds
ASO (MOE)	*Malat1*	5′-GCCAGGCTGGTTATGACTCA-3′	Ss
Toc-HDO (MOE)	*Malat1*	5′-GCCAGGCTGGTTATGACTCA-3′<br>3′-CGG*UCCGACCAAUACUG*AGU-Toc-5′	Ds

Note: orange font indicates locked nucleic acids (LNAs); green font indicates 2′-*O*-methoxyethyl (MOE); red font indicates 2’-O-methyl; black font indicates DNA; *italicized letters* indicate ribonucleic acid (RNA); all phosphorothioate (PS) backbone, unless underlined, is a phosphodiester (PO) linkage.

A, adenine; ASO, antisense oligonucleotide; C, 5-methylcytosine; DNA, deoxyribonucleic acid; G, guanine; HDO, heteroduplex oligonucleotide; RNA, ribonucleic acid; Ss, single strand; Ds, double-strand; T, thymine; Toc, tocopherol; U, uracil.

Table S2. Summary of R^2 values of dose dependent curves

		**R^2 value**	
Target	**DRG**	**ASO**	**Toc-HDO**
Malat1	Lumbar	0.6165	0.5036
Scarb1	Lumbar	0.6613	0.9048

Note: ASO, antisense oligonucleotide; Toc-HDO, α-tocopherol-conjugated heteroduplex oligonucleotide.

Table S3. Evaluation of adverse events

Blood chemistry after a single administration of high-dose ASO or Toc-HDO (*Malat1*)

Blood Chemistry	PBS	ASO	Toc-HDO
Aspartate aminotransferase (IU/l)	66 ± 10.7	36 ± 1.2	43 ± 2.9
Alanine aminotransferase (IU/l)	29 ± 0.6	22 ± 0.8	39 ± 2.4
Total bilirubin (mg/dl)	0.06 ± 0.003	0.05 ± 0.009	0.05 ± 0.005
Direct bilirubin (mg/dl)	0.05 ± 0.003	0.03 ± 0.008	0.03 ± 0.016
Indirect bilirubin (mg/dl)	0.02 ± 0.016	0.03 ± 0.008	0.02 ± 0.012
Triglyceride (mg/dl)	156 ± 14.8	127 ± 6.1	84 ± 11.0
Total cholesterol (mg/dl)	91 ± 2.3	88 ± 1.6	77 ± 2.0
Urea nitrogen (mg/dl)	35.0 ± 1.2	33.9 ± 1.5	31.8 ± 1.1
Creatinine (mg/dl)	0.120 ± 0.001	0.120 ± 0.010	0.120 ± 0.010
Na (mEq/L)	152± 1.2	151 ± 0.4	151 ± 0.4
K (mEq/L)	3.9 ± 0.1	4.90 ± 0.10	3.8 ± 0.2
Albumin (g/dl)	3.6 ± 0.04	3.6 ± 0.06	3.4 ± 0.06

Blood chemistry levels three days after a single administration of 50 mg/kg Toc-HDO

n = 3, data shown are mean values ± SEM.

Blood Chemistry after repeated administration of high-dose ASO or Toc-HDO (*Malat1*)

Blood Chemistry	PBS	ASO	Toc-HDO
Aspartate aminotransferase (IU/l)	58 ± 14.49	64.25 ± 38.76	49 ± 6.37
Alanine aminotransferase (IU/l)	29.75 ± 6.39	29.25 ± 3.40	34.25 ± 5.61
Total bilirubin (mg/dl)	0.04 ± 0.024	0.035 ± 0.012	0.072 ± 0.015
Direct bilirubin (mg/dl)	0.002 ± 0.005	0.002 ± 0.005	0.015 ± 0.017
Indirect bilirubin (mg/dl)	0.037 ± 0.022	0.032 ± 0.009	0.02 ± 0.012
Triglyceride (mg/dl)	90.5 ± 47.47	130.5 ± 23.5	51 ± 6.97
Total cholesterol (mg/dl)	89.5 ± 7.93	98.5 ± 9.0	80 ± 4.83
Urea nitrogen (mg/dl)	36.9 ± 3.4	38.1 ± 2.97	33.5 ± 0.87
Creatinine (mg/dl)	0.115 ± 0.026	0.120 ± 0.016	0.130 ± 0.0
Na (mEq/L)	155.25± 2.2	153.75 ± 0.5	152.25 ± 1.7
K (mEq/L)	4.6 ± 0.51	5.22 ± 0.57	5.65 ± 0.67

Blood chemistry levels three days after repeated administration of 50 mg/kg Toc-HDO

n = 3, data shown are mean values ± SEM.

Note: PBS, Phosphate-buffered saline; ASO, antisense oligonucleotide; Toc-HDO, α-tocopherol-conjugated heteroduplex oligonucleotide.
